# Incorrectly corrected? QT interval analysis in rats and mice

**DOI:** 10.3389/fphys.2022.1002203

**Published:** 2022-10-11

**Authors:** Wesam Mulla, Michael Murninkas, Or Levi, Yoram Etzion

**Affiliations:** ^1^ Cardiac Arrhythmia Research Laboratory, Department of Physiology and Cell Biology, Faculty of Health Sciences, Ben-Gurion University of the Negev, Beer-Sheva, Israel; ^2^ Regenerative Medicine and Stem Cell Research Center, Ben-Gurion University of the Negev, Beer-Sheva, Israel

**Keywords:** rodent cardiac electrophysiology, ECG, QT interval, effective refractory period, action potential duration, rate-adaptation

## Abstract

QT interval, a surrogate measure for ventricular action potential duration (APD) in the surface ECG, is widely used to identify cardiac abnormalities and drug safety. In humans, cardiac APD and QT interval are prominently affected by heart rate (HR), leading to widely accepted formulas to correct the QT interval for HR changes (QT corrected - QTc). While QTc is widely used in the clinic, the proper way to correct the QT interval in small mammals such as rats and mice is not clear. Over the years, empiric correction formulas were developed for rats and mice, which are widely used in the literature. Recent experimental findings obtained from pharmacological and direct pacing experiments in unanesthetized rodents show that the rate-adaptation properties are markedly different from those in humans and the use of existing QTc formulae can lead to major errors in data interpretation. In the present review, these experimental findings are summarized and discussed.

## Introduction

QT interval measurement is an important aspect of any ECG evaluation and interpretation. It has significant clinical importance, as there is a correlation between the QT interval value and the risk of developing ventricular arrhythmias and sudden cardiac death (Viskin, 2009; Lester et al., 2019). The importance of the QT interval did not come to light for several decades after the first description of the morphology of the human ECG by Willem Einthoven in 1885 (Lester et al., 2019). Louise Wolff, an American cardiologist who described the WPW syndrome with Parkinson and White, was probably the first person to measure the QT interval (G Postema and AM Wilde, 2014). However, the clinical importance of the QT interval was not fully understood until further work by Jervell and Lange-Nielsen in the late 1950s (Jervell and Lange-Nielsen, 1957), and by Romano, Gemme, Pongiglione, and Ward in the 1960s (Romano, 1963; Ward, 1964). Several types of long QT syndrome have since been described, and the recognition of the relationship between QT prolongation and serious ventricular arrhythmias has strengthened. Moreover, a series of patients treated with the antiarrhythmic drug quinidine were reported to have syncope due to ventricular tachycardia in the setting of a prolonged QT interval in 1964 (Selzer and Wray, 1964). The morphology of quinidine-induced ventricular tachycardia had a peculiar undulating appearance which in 1966 was termed Torsades de Pointes (TdP) by Desertennes (Dessertenne, 1966). In the subsequent decades, additional classes of medications including antibiotics and psychotropic drugs were linked to TdP and a number of these agents were subsequently withdrawn by the Food and Drug Administration (FDA) (Fung et al., 2001). Drug-induced prolongation of the QT interval is usually caused by the drug’s ability to inhibit IKr, the rapid component of the delayed rectifier potassium current. In humans, this component is encoded by human ether-a-go-go related gene (hERG) (Redfern et al., 2003). In 2007, the FDA formed an internal review team responsible for overseeing the clinical assessment of QT prolongation for all drugs that the agency reviewed, thus assessment of QT prolongation has rapidly become an essential part of the development of new drugs (Darpo, 2010). Although it recommends correcting of the QT interval for HR, it also concedes that such correction can yield misleading results (Food and Drug Administration, 2005). Moreover, literature-based assessments indicate that only non-rodent mammalian models can mimic QT interval prolongation and TdP caused by human therapeutics (Davis, 1998; De Ponti et al., 2001; Webster et al., 2002; Redfern et al., 2003). IKr plays a small role if any in rodents and most studies agree that these models are inappropriate for the study of drug-induced TdP (Hoffmann and Warner, 2006).

## QT interval rate dependence in humans

The QT interval consists of two components: the QRS complex and the T wave, which reflect ventricular depolarization and repolarization, respectively. Duration of the QT interval can vary widely in each individual (Al-Khatib et al., 2003). Many determinants contribute to this variation, including HR, age, sex, autonomic nervous activity, circadian rhythm, drugs, electrolyte variations, myocardial disease, and congenital syndromes (Al-Khatib et al., 2003; Tomaselli Muensterman and Tisdale, 2018). The greatest variation occurs with HR as it is the principal modulator of repolarization duration (Locati et al., 2017). The QT interval dependence on HR reflects the APD dependence on cycle length (CL), a fundamental property of cardiac muscle in humans and large mammals (Franz et al., 1988; Locati et al., 2017). Like APD, QT interval also decreases at shorter CL and prolongs as the CL increases. The kinetics of APD/QT interval adaptation consists of a fast response followed by a gradual course towards a new steady-state value (Franz et al., 1988; Seethala et al., 2011). Mechanisms underlying this adaptive response include inactivation of the L-type calcium current as well as activation of the slow component of the delayed rectifier K^+^ current (Kv7.1/I_ks_). In addition, it appears that mechanisms affecting intracellular Na^+^ accumulation are important determinants of the slow phase of adaptation (Pueyo et al., 2010; O'Hara and Rudy, 2012; Schmitt et al., 2014). ADP and QT interval do not adapt solely on the basis of changes in CL. Exercise and adrenergic stimulation which promote both tachycardia and QT interval shortening are known to induced adaptation beyond that observed during pacing (Seethala et al., 2011). This phenomena involves autonomic stimulation of Kv7.1 (Vyas and Ackerman, 2006). Many QTc formulae have been developed to normalize the QT interval to rate-dependent changes with variable utility in the clinic (Rautaharju et al., 2009; G Postema and AM Wilde, 2014; Locati et al., 2017). The most commonly used correction method in the clinic is the QT Bazett’s formula (G Postema and AM Wilde, 2014; Locati et al., 2017) in which QTc is calculated as the QT interval in seconds divided by the square root of the preceding CL in seconds (Bazett, 1920). When HR is particularly fast or slow, the Bazett’s formula may over or underestimate the baseline QT, respectively. However, regardless of this limitation it still remains the current standard in clinical practice (G Postema and AM Wilde, 2014; Locati et al., 2017).

## Myocardial repolarization in rats and mice

Although the overall principles of myocardial excitation are the same in all mammalian species, the role of repolarizing currents markedly differ between humans and rodents (Boukens et al., 2014). This is presumably dictated by the great variations in HR and HR modulation among species. Humans have a resting HR of ≈60 bpm, whereas rats and mice have a HR of on average 6 and 10 times higher, respectively (Kaese and Verheule, 2012; Milani-Nejad and Janssen, 2014; Konopelski and Ufnal, 2016). In addition, while rats and mice can increase their HR by around 40%–50% and 30%–40% respectively, humans can increase their HR by up to 300% (Milani-Nejad and Janssen, 2014; Janssen et al., 2016). These differences require repolarizing K^+^ ionic currents with different kinetics in order to adapt the APD appropriately. In rats and mice, the major repolarizing K^+^ ionic currents are the transient outward K^+^ current (I_to_) and ultra-rapid potassium current (I_kur_), and the ventricular APD has a triangular shape, with short repolarization and no clear plateau phase (Watanabe et al., 1983; Varró et al., 1993; Knollmann et al., 2001). Importantly, most rodent studies involve direct pacing under anesthesia, or *ex-vivo*/*in-vitro* preparations, overlooking the effects of autonomic stimulation. Indeed, there is some evidence that IKs mediates repolarization in rat cardiomyocytes under β-adrenergic stimulation (Xu et al., 2016). In addition, among mice, the most phosphorylated protein upon β1-adrenergic receptor activation of the heart is Kv7.1 (Lundby et al., 2013). Nevertheless, the *in-vivo* effect of I_Ks_ on the QT of mice is questionable considering the notable QT prolongation observed in response to β-adrenergic stimulation (Speerschneider and Thomsen, 2013). Adrenergic stimulation also resulted in phosphorylation of I_Na_ (Nav1.5), an additional potential mechanism by which changes in autonomic balance may affect repolarization duration (Lundby et al., 2013). Thus, the mechanisms governing APD in the unanesthetized rodent seem to be far more complex than I_to/_I_Kur_ -dependent repolarization.

## Rate-adaptation studies of ADP and QT interval in rodents

As mentioned above the basic electrophysiological components governing ADP in the rodent myocardium are relatively well known. Still, *in-vivo* rate-dependence of the APD and QT are rather poorly defined in these species. As already mentioned, one possible explanation for this is the technical challenge in performing advanced EP studies in unanesthetized rodents. In addition, results may vary substantially depending on the used methodology (i.e., type of anesthesia, type of perfused solution) and indeed, data regarding the electrophysiological properties of rodents show marked variations in the literature (Kaese and Verheule, 2012). Although the determinants of the rate-dependence of APD differ between small rodents and humans (as described above) there are various publications supporting the notion that typical rate-adaptation still exists in rodents (Table 1). However, various publications have demonstrated no rate-adaptation or even atypical rate-adaptation (i.e., increased APD at shorter CL). This variability raise the question whether discrepancies between studies might be secondary to differences in techniques (e.g., techniques that interfere with endogenous autonomic modulation such as anesthetics, large doses of exogenous catecholamines, overcontrolling for circadian rhythm). In any case, these discrepancies stresses the importance of obtaining data from unanesthetized rodents under physiological conditions in order to arrive at reliable conclusions. Another major challenge and source of uncertainty in evaluations of the relationship between HR and repolarization of rodent and particularly mice is identification of the end of the T wave. Because of the high HRs, motion artifact, and other sources of signal noise with telemetry devices, obtaining clean ECG signals in conscious rodents can be particularly challenging. As well, the end of the negative murine T wave is more subtle and therefore elusive to automated software detection than in humans and other mammals with more overt, positive T waves.

**TABLE 1:** Summary of studies in which QT interval, ERP and APD rate relation were evaluated in rats and mice.

Experiment Parameter		Unanesthetized			Anesthetized			*Ex-vivo*/*In-vitro*		
		Pacing	Pharmacologic	Physiologic	Pacing	Pharmacologic	Physiologic	Pacing	Pharmacologic	Physiologic
**Rats**	QT interval	**↔** Mulla et al. (2018)	**↔** Wallman et al. (2021)	**↔** Fein et al. (1991), Adeyemi et al. (2020), Wallman et al. (2021)	**↔** Hayes et al. (1994)	**↔** Kmecova and Klimas (2010)	**↔** Ohtani et al. (1997)			
	Atrial ERP	**↔** Murninkas et al. (2020)	**↔** Etzion et al. (2008)		**↔** Etzion et al. (2008)					
					**↓** Li et al. (2020)					
	Ventricular ERP	**↔** Mulla et al. (2018)			**↑↔** Ypma (1972)			**↑ ↔** Ypma (1972)		
	Atrial APD							**↔↓** Huang et al. (2006)		**↔** Couch et al. (1969)
	Ventricular APD				**↔** Rapuzzi and Rindi (1967)			**↑** Shimoni et al. (1994), Shimoni et al. (1995), Shigematsu et al. (1997), Fauconnier et al. (2003)	**↔** Sakatani et al. (2006)	**↔** Couch et al. (1969)
								**↔** Blesa et al. (1970), Pucelik et al. (1982), Shigematsu et al. (1997), Pacher et al. (1999), Benoist et al. (2011), Benoist et al. (2012), Walton et al. (2013), Hardy et al. (2018)	**↔↓** Howlett et al. (2022)	
								**↑↓** Watanabe et al. (1983)	**↓** Wang and Fitts (2017)	
								**↔↓** Keung and Aronson (1981), Howlett et al. (2022)		
								**↓** Payet et al. (1981), Watanabe et al. (1983), Wang and Fitts (2017)		
**Mice**	QT interval	**↔** Mulla et al. (2018)	**↔** Roussel et al. (2016)	**↔** Roussel et al. (2016), Schroder et al. (2021), Warhol et al. (2021)	**↔** Speerschneider and Thomsen (2013)	**↑** Speerschneider and Thomsen (2013)			**↔** Joyce et al. (2021)	
				**↓** Mitchell et al. (1998) **↑↔↓** Sudhir et al. (2020)		**↔** Warhol et al. (2021)				
	Atrial ERP				**↔** Etzion et al. (2008)			**↔** Syeda et al. (2016), Obergassel et al. (2021)		
					**↓** Berul et al. (1996)					
	Ventricular ERP	**↔** Mulla et al. (2018)			**↓** Berul et al. (1996)			**↔** Waldeyer et al. (2009)		
	Atrial APD							**↔** Syeda et al. (2016)		
								**↓** Knollmann et al. (2007), Obergassel et al. (2021)		
	Ventricular APD				**↔↓** Nuyens et al. (2001)			**↔** Wagner et al. (2006), Kulkarni et al. (2018), Joyce et al. (2021)	**↔** Sudhir et al. (2020), Joyce et al. (2021)	**↑** Francis Stuart et al. (2018)
								**↓** Knollmann et al. (2007), Sabir et al. (2008), Waldeyer et al. (2009), Mulla et al. (2018)	**↓** Francis Stuart et al. (2018)	

Typical rate adaptation ↓ Flat rate adaptation ↔ Atypical rate adaptation ↑

## Discussion

**FIGURE 1:**
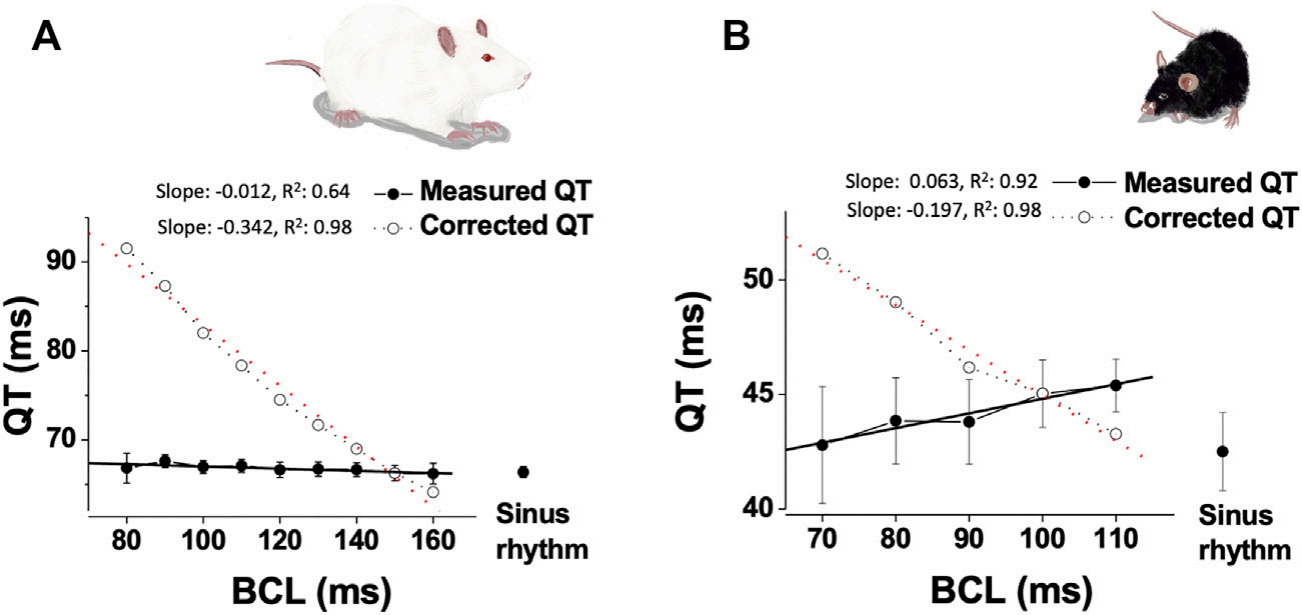
Absence of QT interval dependence on HR in paced unanesthetized rodents. **(A)**. Data from instrumented, unanesthetized male SD rats that were subjected to atrial pacing at different CL (*n* = 10): Mean ± SE of measured QT and calculated QTc (based on the formula of Kmecova and Klimas (Kmecova and Klimas, 2010)), plotted as a function of CL. Linear regression data (slope, R^2^) are presented for both QT and calculated QTc. **(B)**. Data from instrumented, unanesthetized male C57BL/J mice that were subjected to atrial pacing at different CL (*n* = 9): Mean ± SE of measured QT and calculated QTc [based on the formula of Mitchell et al. (1998)], plotted as a function of CL. Linear regression data (slope, R^2^) are presented for both QT and calculated QTc. Experimental findings were adapted from Mulla et al. (2018).

The dependence of QT interval of HR is debatable in rats and mice (Hayes et al., 1994; Ohtani et al., 1997; Mitchell et al., 1998; Kmecova and Klimas, 2010; Speerschneider and Thomsen, 2013; Roussel et al., 2016; Mulla et al., 2018). For example, Mitchell et al. (Mitchell et al., 1998) used the natural daily variation in HR in unanesthetized mice and found a strong correlation between the QT interval and HR, where slower HRs were associated with longer QT intervals. In contrast, Roussel et al. (Roussel et al., 2016) examined the same natural daily variation in HR as well as changes induced by tachycardic agents (norepinephrine or nitroprusside) and concluded that increased HR was not associated with apparent shortening of the QT interval. Nevertheless, at least for the circadian data it might be possible that analysis of light and dark phases separately for QT-RR relationships may over-control for endogenous changes in rate and repolarization mediated by circadian fluctuations in rhythm and autonomic modulation. Sudhir et al. (Sudhir et al., 2020) recently explored adaptation of QT to physical stress in mice overexpressing SUR2A, a regulatory subunit of sarcolemmal ATP-sensitive K + (K_ATP_) channels. Although the results show highly complex pattern of changes over time in both transgenic and control mice, only corrected QT intervals (using Mitchell’s formula) are presented in the study, limiting ability to evaluate the effects of exercise on the native QT interval. In rats, a QT interval correction formula suggested by Kmecova and Klimas (Kmecova and Klimas, 2010) was validated using pharmacological manipulations affecting HR. Interestingly, adrenergic stimulation with isoproterenol as well as selectively manipulation of the HR by ivabradine, did not affect the QT interval in this study. Technical challenges largely limited direct pacing experiments as a mean of evaluating QT rate-dependence in rodents. However, Mulla et al. (2018) managed to explore the QT interval of freely moving rats and mice during atrial pacing at various CL values through a unique chronically implanted device. The findings of this work indicated absence of conventional rate-adaptation of the QT interval over a wide range of physiologically relevant frequencies. Moreover, ventricular ERP (a surrogate of ventricular APD) also demonstrated absence of typical rate-dependence. Calculating the QTc interval according to the formulae suggested by Kmecova and Klimas (Kmecova and Klimas, 2010) for rats and by Mitchell et al. (1998) for mice, resulted in marked difference between the measured QT interval and the QTc interval for a wide range of atrial pacing rates in both species (Figure 1). At the present, it is hard to conclude what is the optimal way of QT correction in rodent and if correction is required at all. However, it appears that the existing and widely use correction formulas can introduce marked errors. This issue seems specifically relevant for Mitchell formula for mice that appears to overcorrect QT, indicating a great need for a better QT correction formula for mice. Overall, we suggest that any correction formula used should be validated in that species under baseline conditions using comparable analytic methods and measurement techniques as those applied during/after experimental treatments. As well, we suggest that as a standard, corrected QT results should be presented along with those for uncorrected QT. Importantly, to the best of our knowledge direct data on the relationship between QT and HR in the unanesthetized Guinea pig are lacking in literature. Given the existence of I_kr_ and I_ks_ in the myocardium and their broad utility for QT prolongation studies, we believe that understanding the precise HR-QT relationship of this animal model in future studies will be of high value as well.

In conclusion, conflicting data still exists regarding the dependence between QT interval and HR in rodents. Multiple physiological and technical complexities and challenges prevents clear conclusions regarding this issue based on the currently available data. However, large body of evidence support the notion that the extensive use of existing correction formulae may introduce significant errors and thus further and more systematic exploration of this issue would be of high value.
